# ﻿Mealybugs (Hemiptera, Coccomorpha, Pseudococcidae) on parasitic plants (Loranthaceae) in Indonesia with description of a new species and a new country record

**DOI:** 10.3897/zookeys.1167.106012

**Published:** 2023-06-15

**Authors:** Agustin Zarkani, Ariffatchur Fauzi, Dwinardi Apriyanto, Mehmet Bora Kaydan

**Affiliations:** 1 Department of Plant Protection, Faculty of Agriculture, University of Bengkulu, 383711, Bengkulu, Indonesia University of Bengkulu Bengkulu Indonesia; 2 Biotechnology Development and Research Centre, Çukurova University, 01250, Adana, Turkiye Çukurova University Adana Turkiye

**Keywords:** Biodiversity, identification key, new record, parasitic plant, pests, Sternorrhyncha, taxonomy

## Abstract

Parasitic plants have been known to be attacked by insect pests since ancient times. However, little is known about the mealybug (Hemiptera, Coccomorpha, Pseudococcidae) fauna associated with them. A series of surveys of mealybugs found on Loranthaceae, a semi-parasitic plant family, was conducted in several places in Bengkulu Province, southern Sumatra, Indonesia. In the study, 55 mealybug specimens were collected, consisting of eight species belonging to five genera, namely *Chorizococcus* McKenzie (1 species), *Dysmicoccus* Ferris (2 species), *Ferrisia* Fullaway (1 species), *Planococcus* Ferris (3 species) and *Pseudococcus* Westwood (1 species). *Chorizococcusozeri* Zarkani & Kaydan, **sp. nov.** is new to science, whilst *Planococcusbagmaticus* Williams represents the first record in Indonesia. In addition, the mealybugs *Dysmicoccuslepelleyi* (Betrem), *Dysmicoccuszeynepae* Zarkani & Kaydan, *Ferrisiadasylirii* (Cockerell), *Planococcuslilacinus* (Cockerell) and *Pseudococcusjackbeardsleyi* Gimpel & Miller are newly recorded from plants of the family Loranthaceae. Figures and illustrations of mealybug species with a taxonomic key to Asian *Chorizococcus* and a new country record based on morphological characters are also updated.

## ﻿Introduction

Loranthaceae is a primitive family of parasitic plants which are photosynthetic xylem feeders and cannot exist independently of the host plant (Musselman and Press 1995). Parasitic plants often severely reduce agricultural plant production, which impact the plant community (Press and Phoenix 2005). Their productivity and populations are therefore co-dependent on both the quality of the plant hosts that they parasitize and the strength of competition from neighboring plants (Pennings and Callaway 2002). In addition, a decrease in the quality of the host plants will affect organisms at other trophic levels such as herbivores and pollinators, and ultimately also affect the conditions of the abiotic environment, including having an impact on the nutrient cycles, groundwater relations, local temperature, and atmospheric CO_2_ concentrations (Press and Phoenix 2005).

Just as non-parasitic plants have been attacked by insect pests for many generations, parasitic plants have also been known to be attacked by insect pests since ancient times. However, little information about the mealybug fauna (Hemiptera, Coccomorpha, Pseudococcidae) associated with parasitic plants is known. According to the scale insect database ScaleNet (url:scalenet.info/), 18 species of mealybugs (Hemiptera, Pseudococcidae) have been reported to be associated with Loranthaceae worldwide, namely *Anisococcusparasitus* Williams & Granara de Willink, *Coccidohystrixinsolita* (Green), *Dysmicoccusambiguous* (Morrison), *Dysmicoccusdebregeasiae* (Green), *Dysmicoccusviticis* (Green), *Erioidesrimulae* Green, *Exallomochlushispidus* (Morrison), *Macrocepicoccusloranthi* Morrison, *Nipaecoccuskosztaraborum* Williams & Granara de Willink, *Nipaecoccusnipae* (Maskell), *Paraputoloranthi* (Matile-Ferraro), *Planococcusbendovi* Williams, *Planococcuskenyae* (Le Pelley), *Porococcuscoxatus* Ferris, *Porococcuspergandei* Cockerell, *Porococcustinctorius* Cockerell, *Pseudococcuscomstocki* (Kuwana) and *Pseudococcusviburni* (Signoret) (García-Morales et al. 2016). In Indonesia, there are five species that have been reported that are associated with Loranthaceae, namely *C.insolita*, *D.debregeasiae*, *E.hispidus*, *P.bendovi*, and *P.viburni* (García-Morales et al. 2016; Zarkani et al. 2021, 2022; Sartiami et al. 2022).

For decades, the study of parasitic plants focused mainly on genetic variability, chemical contents, and their impact on their host plants. In this study we report several species of mealybugs found on Loranthaceae in Indonesia and provide an updated list of parasitic plant-feeding scale insects in the world. These specialized pests could be evaluated as natural control agents of parasitic plants in the future.

## ﻿Materials and methods

Adult mealybug females were collected from a series of sampling occasions on leaves, trunk, and branches of Loranthaceae trees in Bengkulu Province, southern Sumatra, Indonesia from March to December 2022. The sampling sites are at an altitude of 0–1100 m above sea level. The specimens were mounted and preserved in slides and identified to genus level. The slide mounting was carried out under a binocular dissection microscope, LEICA EZ4HD by using the method described Kosztarab and Kozár (1988).

Species identifications were made by observing the specific features of the mealybug species using a phase-contrast compound microscope (LEICA DM2700) and were identified using the keys in Williams and Watson (1988), Williams and Granara de Willink (1992) and Williams (2004). The morphological parameters used are those used by Williams and Granara de Willink (1992), Williams (2004) and Zarkani et al. (2023). The body width and length were measured in mm which is the largest transverse measurement perpendicular to the longitudinal axis and the longest longitudinal, respectively. Other measurements are given in μm in which the standardized measurements of anatomical features, for example, antennal segments, leg segments, anal ring, pores are given. Antennae length is the sum of all segments of the antennae. Leg length is the sum of the lengths of the trochanter + femur, tibia + tarsus, and claw. In the taxonomic illustrations, the dorsal morphology is shown on the left side whilst the ventral morphology is shown on the right side. Type specimens of the genus and species described are deposited in the Mealybugs Museum, Department of Plant Protection, Faculty of Agriculture, University of Bengkulu, Bengkulu, Indonesia (MMUB).

## ﻿Results and discussion

A series of surveys carried out in southern Sumatra on Loranthaceae resulted in the collection of 55 mealybug specimens consisting of eight species belonging to five genera. The identified species belong to the genera *Chorizococcus* McKenzie (1 species), *Dysmicoccus* Ferris (2 species), *Ferrisia* Fullaway (1 species), *Planococcus* Ferris (3 species) and *Pseudococcus* Westwood (1 species). One species is new to science, *Chorizococcusozeri* Zarkani & Kaydan, whilst another, *Planococcusbagmaticus* Williams is a newly recorded in Indonesia. In addition, this is the first report of the genus *Chorizococcus* attacking Loranthaceae worldwide. Furthermore, the mealybugs *Dysmicoccuslepelleyi* (Betrem), *Dysmicoccuszeynepae* Zarkani & Kaydan, *Ferrisiadasylirii* (Cockerell), *Planococcuslilacinus* (Cockerell) and *Pseudococcusjackbeardsleyi* Gimpel & Miller were found for the first time associated with Loranthaceae in the world.

Currently, a total of 18 mealybug species (Hemiptera, Pseudococcidae) have been reported on plants of the family Loranthaceae worldwide (García-Morales et al. 2016); hence within this study, the number of mealybug species on these parasitic plants is increased to 26 species. The species marked with an asterisk (*) below are recorded for the first time from Indonesia. Furthermore, the species that are new host records on Loranthaceae worldwide are indicated with a plus mark (+).

### ﻿ Chorizococcus

Taxon classificationAnimaliaHemipteraPseudococcidae

McKenzie

E411238F-A248-58A8-AB92-35228CD7FA20

#### Type species.

*Chorizococcuswilkeyi* McKenzie, by original designation.

#### Genus diagnosis.

(adapted from Williams, 2004). Body of adult female membranous, varying in shape from elongate oval with almost parallel sides, to broadly oval. With 1–5 pairs of cerarii present on posterior segments of abdomen and sometimes a pair on head also, each cerarius bearing 2 conical setae; auxiliary setae absent from cerarii anterior to anal lobe pair. Oral rim ducts, sometimes of 2 sizes, present on dorsum and frequently also on venter. Oral collar tubular ducts usually present, at least on venter; if present on dorsum, then restricted to margins. Antennae each normally with 7 or 8 segments. Legs well developed, with translucent pores usually present, at least on hind coxae. Claw normally stout, without a denticle. Tarsal digitules minutely knobbed. Multilocular disc pores present on venter, rarely found on dorsum. Circulus present or absent, when present usually divided by an intersegmental line. Anal ring normal, bearing 6 setae. Anterior and posterior ostioles present.

### ﻿ Chorizococcus ozeri

Taxon classificationAnimaliaHemipteraPseudococcidae

Zarkani & Kaydan sp. nov.

19122720-D2C1-5694-80A3-DFE6B27E324F

https://zoobank.org/BB94C9AB-2715-4C87-8DAA-BAABC0A5DC72

Figs 1 , 2

#### Material examined.

(all deposited at MMUB). ***Holotype*.** Adult female, left label: AZ1204, 13.vii.2022, Indonesia, Sumatra, Bengkulu, ex *Loranthus* sp., 03°45'10″S, 102°16'59″E, 120 m a.s.l.; right label: *Chorizococcusozeri* Zarkani & Kaydan, 3 ♀♀, coll. A. Zarkani, det. M.B. Kaydan. The holotype specimen is ringed with red ink on the coverslip. ***Paratypes*.** 3 ♀♀, Indonesia: (AZ1205), same data as holotype; 3 ♀♀, AZ206, Sumatra, Bengkulu on semi-parasitic plant, *Loranthus* sp. (Loranthaceae), living on avocado (*Perseaamericana* Mill.), 03°45'10″S, 102°16'59″E, 13.vii.2022, coll. A. Zarkani.

#### Description of adult female.

**Figure 1.**
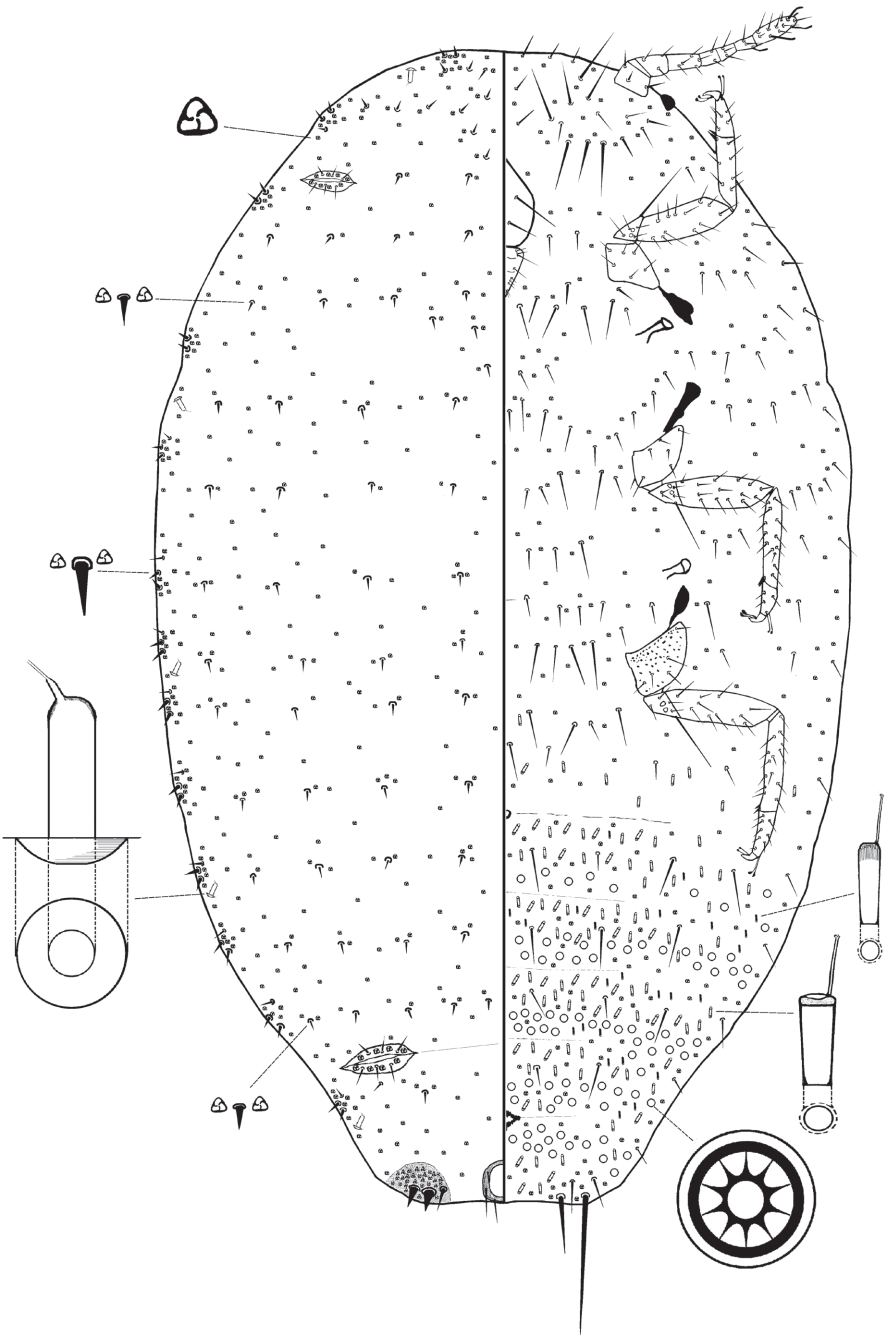
Adult female of *Chorizococcusozeri* Zarkani & Kaydan, sp. nov., holotype.

***Appearance in life*** (Fig. 1). Adult females produce a powdery white wax covering the dorsal surface of their bodies. Living on parasitic roots, branches, leaves, and flowers of its host plant, commonly attended by ants of the genus *Dolichoderus* Lund.

**Figure 2.**
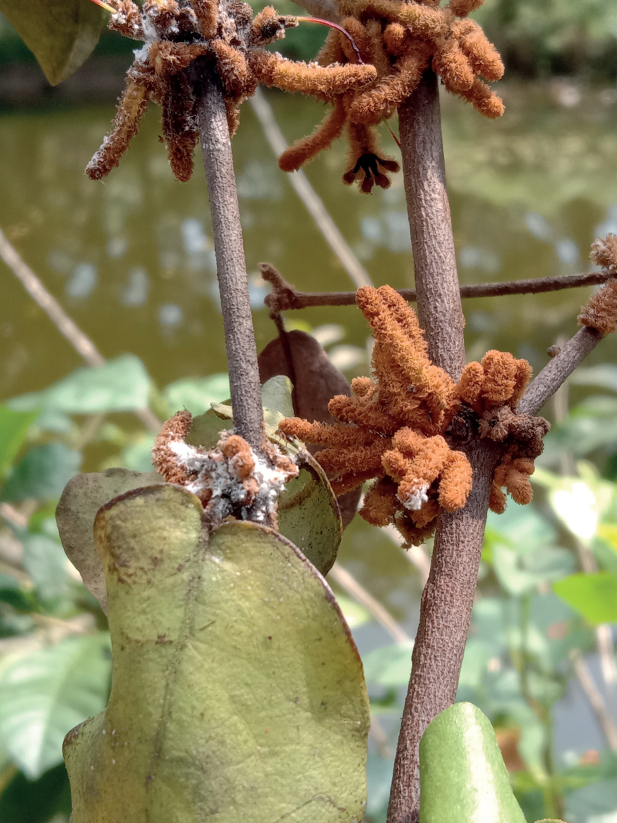
An aggregation of *Chorizococcusozeri* Zarkani & Kaydan, sp. nov., on a semi-parasitic plant, *Loranthus* sp. (Loranthaceae), living on an avocado (*Perseaamericana* Mill.), in Bengkulu Province, Sumatra (03°45'10″S, 102°16'59″E).

***Slide-mounted adult female*** (based on the holotype and 3 paratypes) (Fig. 2). Body oval, 2.25–2.28 mm long, 1.40–1.50 mm wide. Eyes situated on margins, each 17.5–30 μm wide. Antenna 7 segmented, 370–380 μm long, with 4 fleshy setae, each 20–25 μm long; apical segment 87.5–92.5 μm long, 30.0–32.5 μm wide, with apical seta 30.0–32.5 μm long. Clypeolabral shield 112.5–132.5 μm long, 87.5–100 μm wide. Labium 3 segmented, 67.5–87.5 μm long, 67.5–75.0 μm wide. Anterior spiracles each 57.5–75.0 μm long, 30.0–42.5 μm wide across atrium; posterior spiracles each 62.5–75.0 μm long, 32.5–37.5 μm wide across atrium. Circulus rounded or quadrate, 11.3–12.5 μm wide. Legs well developed; segment lengths for each posterior leg: coxa 125–175 μm, trochanter + femur 237.5–307.5 μm, tibia + tarsus 225–300 μm, claw 25.0–27.5 μm. Ratio of length of tibia + tarsus to trochanter + femur, 0.95–0.98: 1; ratio of length of tibia to tarsus, 1.81–2.16: 1; ratio of length of trochanter + femur to greatest width of femur, 3.8–4.39: 1; coxa with translucent pores, femur and tibia without translucent pores. Tarsal digitules capitate, each 37.5–50.0 μm long. Claw digitules capitate, each about 20.0–22.5 μm long. Both pairs of ostioles present, anterior ostioles each with a total for both lips of 6–11 trilocular pores and without setae; posterior ostioles each with a total for both lips of 6–9 trilocular pores and without setae. Anal ring about 80.0–87.5 μm wide, bearing 6 setae, each seta 87.5–100.0 μm long.

***Dorsum*.** Derm membranous, with 13 pairs of cerarii around body margin, each cerarius with enlarged conical setae set up in 2 rows and each with 1 auxiliary setae. Each anal lobe cerarius set on membranous cuticle and containing 2 enlarged conical setae, each 15–25 μm long, plus 8–10 trilocular pores and 2–3 hair-like auxiliary setae, each about 10–12 μm long. Dorsal setae conical, thinner than most cerarian setae, each 5.0–7.5 μm long, median setae 10.0–12.5 μm long, scattered throughout dorsum. Trilocular pores, each 2.5–3.8 μm in diameter, scattered. Multilocular disc pores and tubular ducts absent.

***Venter*.** Setae flagellate, each 87.5–122.5 μm long, longest setae located medially on head. Apical setae on anal lobes unusually short, each 125.0–127.5 μm long. Multilocular disc pores, each 6.25–7.5 μm in diameter, present on abdominal segments, distributed as follows (mean numbers): IV: 18, V: 42, VI: 44, VII: 28, and VIII: 18. Trilocular pores, each 2.5–3.8 μm across, scattered throughout venter. Oral collar tubular ducts with two types: large type each 7.5–10.0 μm long, 2.5–3.1 μm wide, present one on each marginal abdominal segments V–VIII and mesothorax, and more abundantly on mid areas of thorax.

#### Comments.

*Chorizococcusozeri* is most similar to *Chorizococcussrinagaricus* Williams in having no oral rim tubular ducts on the venter; dorsal rim tubular ducts few, present mainly either in medial areas or marginal areas. However, *C.ozeri* can be distinguished from *C.srinagaricus* in having (character states for *C.srinagaricus* given in parentheses): (i) oral rim tubular ducts present mainly in marginal areas of dorsum (mainly in medial areas of dorsum); (ii) oral collar tubular ducts absent on dorsum (present); (iii) ventral oral collar tubular ducts present around abdomen only (present on entire body surface); (iv) large discoidal pores absent from venter (present); and (v) translucent pores on hind coxa present (absent).

It is also similar to *Chorizococcussorgi* Williams in lacking oral collar tubular ducts entirely from ventral margins of head and thorax; multilocular disc pores and oval collar tubular ducts absent from the area lateral to each first coxa. However, *C.ozeri* can be distinguished from *C.sorgi* in having (character states for *C.sorgi* given in parentheses): (i) cerarii confined to anal lobes only (present on at least 3 posterior cerarii); (ii) no oral rim tubular ducts on venter (oral rims present on venter); and (iii) multilocular disc pores in two rows on venter (in one row).

#### Etymology.

This species is named after Emin Ozer (Business Sustainability Lead Turkiye, Syngenta Tarim San. ve Tic. A.Ş., Yeni Mahalle 87071 Sk. Bozkurtlar Rezidans No: 52 K/D:12/25, Seyhan – Adana / TURKIYE), one of the best partners and mentor of the Kaydan’s Laboratory.

#### Host plants.

*Loranthus* sp. (Loranthaceae) (Fig. 2).

#### Distribution.

Indonesia (Sumatra, Bengkulu Province).

### ﻿Key to adult female *Chorizococcus* found in southern Asia

**Table:** 

1(0)	Cerarii confined to anal lobes only	**2**
–	Cerarii present on at least 3 posterior segments of abdomen and sometimes on head	**5**
2(1)	Oral rim tubular ducts of 2 sizes present on venter, in considerable number around margins and submarginal areas	***C.kandyensis* (Green)**
–	Oral rim tubular ducts of 1 size only, either absent from venter or present in small number only, on margins and medial areas	**3**
3(2)	Ventral oral rim tubular ducts present, scattered on head, thorax and abdomen. Dorsal oral rim tubular ducts numerous laterally, relatively few present medially	***C.graminis* Khalid & Shafee**
–	Ventral oral rim tubular ducts absent. Dorsal oral rim tubular ducts few, present mainly either in medial areas or margin areas	**4**
4(1)	Dorsal oral rim tubular ducts present mainly in medial areas. Dorsal and ventral oral collar tubular ducts present throughout dorsum and venter. Large discoidal pores, some almost as large as multilocular disc pores, present on venter. Translucent pores on hind coxa apparently absent	***C.srinagaricus* Williams**
–	Dorsal oral rim tubular ducts present mainly in margin areas. Dorsal oral collar tubular ducts absent. Ventral oral collar tubular ducts present around abdomen only. Large discoidal pores absent from venter. Translucent pores on hind coxa present	***C.ozeri* Zarkani & Kaydan**
5(1)	Ventral multilocular disc pores present around vulva only, numbering 2–4	***C.alami* Khalid & Shafee**
–	Ventral multilocular disc pores present across abdominal segments, at least as far forward as abdominal segment IV, numbering more than 10	**6**
6(5)	Oral collar tubular ducts present on ventral margins of head and thorax; a group of tubular ducts associated with 1 or 2 multilocular disc pores situated lateral to each first coxa	***C.irretitus* Williams**
–	Oral collar tubular ducts absent entirely from ventral margins of head and thorax; multilocular disc pores and oval collar tubular ducts absent from lateral to each first coxa	***C.sorgi* Williams**

### ﻿*Dysmicoccus* Ferris

#### ﻿ Dysmicoccus lepelleyi

Taxon classificationAnimaliaHemipteraPseudococcidae

(Betrem)*

316E2654-2DBC-5E5B-89DF-31883B463711

##### Material examined.

Indonesia, Sumatra, Bengkulu Province, North Bengkulu District, Kemumu, on *Loranthus* sp. (Loranthaceae), living on cacao (*Theobromacacao* L.), 600 m a.s.l, 03°26'00″S, 102°15'15″E, 11.v.2022, coll. A. Zarkani (AZ983-984), 6 ♀♀.

##### Comments.

The species is polyphagous on 25 plant genera within 17 families: Anacardiaceae, Annonaceae, Arecaceae, Asparagaceae, Clusiaceae, Euphorbiaceae, Fagaceae, Malvaceae, Meliaceae, Moraceae, Musaceae, Myrtaceae, Rubiaceae, Rutaceae, Sapindaceae, Sapotaceae, and Zingiberaceae (García-Morales et al. 2016; Zarkani et al. 2021). In Indonesia, *D.lepelleyi* has been recorded previously from Java, Lombok and Sumatra (Ben-Dov 1994; Williams 2004). It is also found in neighboring countries such as Cambodia, Malaysia, Singapore, Thailand, and Vietnam (Williams 2004; García-Morales et al. 2016; Zarkani et al. 2021).

#### ﻿ Dysmicoccus zeynepae

Taxon classificationAnimaliaHemipteraPseudococcidae

Zarkani & Kaydan*

E30EA0FC-F325-5A11-A4A2-8A7EEF343356

##### Material examined.

Indonesia, Sumatra, Bengkulu Province, Seluma District, Napal Jungur, on *Loranthus* sp. (Loranthaceae), living on Jengkol (*Pithecellobiumlobatum* Benth), 205 m a.s.l., 03°57'12″S, 102°30'09″E, 5.iii.2023, coll. A. Zarkani (AZ1248), 3 ♀♀.

##### Comments.

*Dysmicoccuszeynepae* is a polyphagous species found on ornamental plants and tropical fruits such as *Duriozibethinus* Murray (Malvaceae), *Lansiumparasiticum* Corrêa (Meliaceae), *Manilkarazapota* Linnaeus (Sapotaceae) and *Coffearobusta* Lindl. ex de williamson (Rubiaceae) (Zarkani et al. 2023). The species is known to have some special features such as small legs, no multilocular disc pores and oral collar tubular ducts on dorsum, with a few multilocular disc pores without oral collar tubular ducts on venter and having translucent pores on the hind coxa and femur. This is the first report of *Dysmicoccus* infestation on Loranthaceae worldwide.

### ﻿*Ferrisia* Fullaway

#### ﻿ Ferrisia dasylirii

Taxon classificationAnimaliaHemipteraPseudococcidae

(Cockerell)*

F70214EE-F17A-5D31-9255-10B24310C3F6

##### Material examined.

Indonesia, Sumatra, Bengkulu Province, Bengkulu City, Teluk Segara, on *Loranthus* sp. (Loranthaceae), living on cucumber tree (*Averrhoabilimbi* L.), 30 m a.s.l., 03°47'18″S, 102°15'15″E, 12.vi.2022, coll. A. Zarkani (ΑΖ1080-1081), 3 ♀♀.

##### Comments.

The species is polyphagous on ornamental plants and fruits belonging to 30 plant families and 54 genera. It is cosmopolitan, being found in 24 countries; in Indonesia it was first recorded from Bengkulu Province, Southern Sumatra on *Duriozibethinus* Murray (Malvaceae), *Gliricidiasepium* (Jacq.) (Fabaceae), *Hibiscus* spp. (Malvaceae), *Psidiumguajava* L. (Myrtaceae), *Solanumtorvum* Swartz (Solanaceae) and *Theobromacacao* L. (Malvaceae) (Zarkani et al. 2020).

### ﻿*Planococcus* Ferris

#### ﻿ Planococcus bagmaticus

Taxon classificationAnimaliaHemipteraPseudococcidae

Williams*+

78A0EDA9-629A-510D-A3B2-F92955964562

Fig. 3

##### Material examined.

Indonesia, Sumatra, Bengkulu Province, Seluma City, Air Periukan, on *Loranthus* sp. (Loranthaceae), living on cacao tree (*Theobromacacao* L.), 30 m a.s.l., 04°01'37″S, 102°24'50″E, 08.vii.2022, coll. A. Zarkani (AZ1112-1114), 6 ♀♀.

##### Comments.

The holotype and paratypes specimens were recorded from Nepal, all in a single slide and deposited at Entomological Institute, Hokkaido University, Sapporo, Japan (HUSJ) and Natural History Museum, United Kingdom, London (BMNH), respectively. It was originally recorded from *Trachelospermum* sp. (Apocynaceae) (Williams 2004). This is the second report of the species after Takagi collected the species from Kathmandu Valley, Bagmati, Godavari-Nepal in 1975 (Williams 2004). It is the only known species of *Planococcus* in southern Asia with dorsal multilocular disc pores. The species is closed to *Planococcusepulus* De Lotto described from Kenya which also has dorsal multilocular disc pores, but *P.epulus* possesses dorsal transverse rows of oral collar tubular ducts, whereas in *P.bagmaticus*, all dorsal oral collar tubular ducts are restricted to small lateral groups on abdominal segments VI and VII. The species was sometimes found mixed with specimens of *P.jackbeardsleyi*.

**Figure 3.**
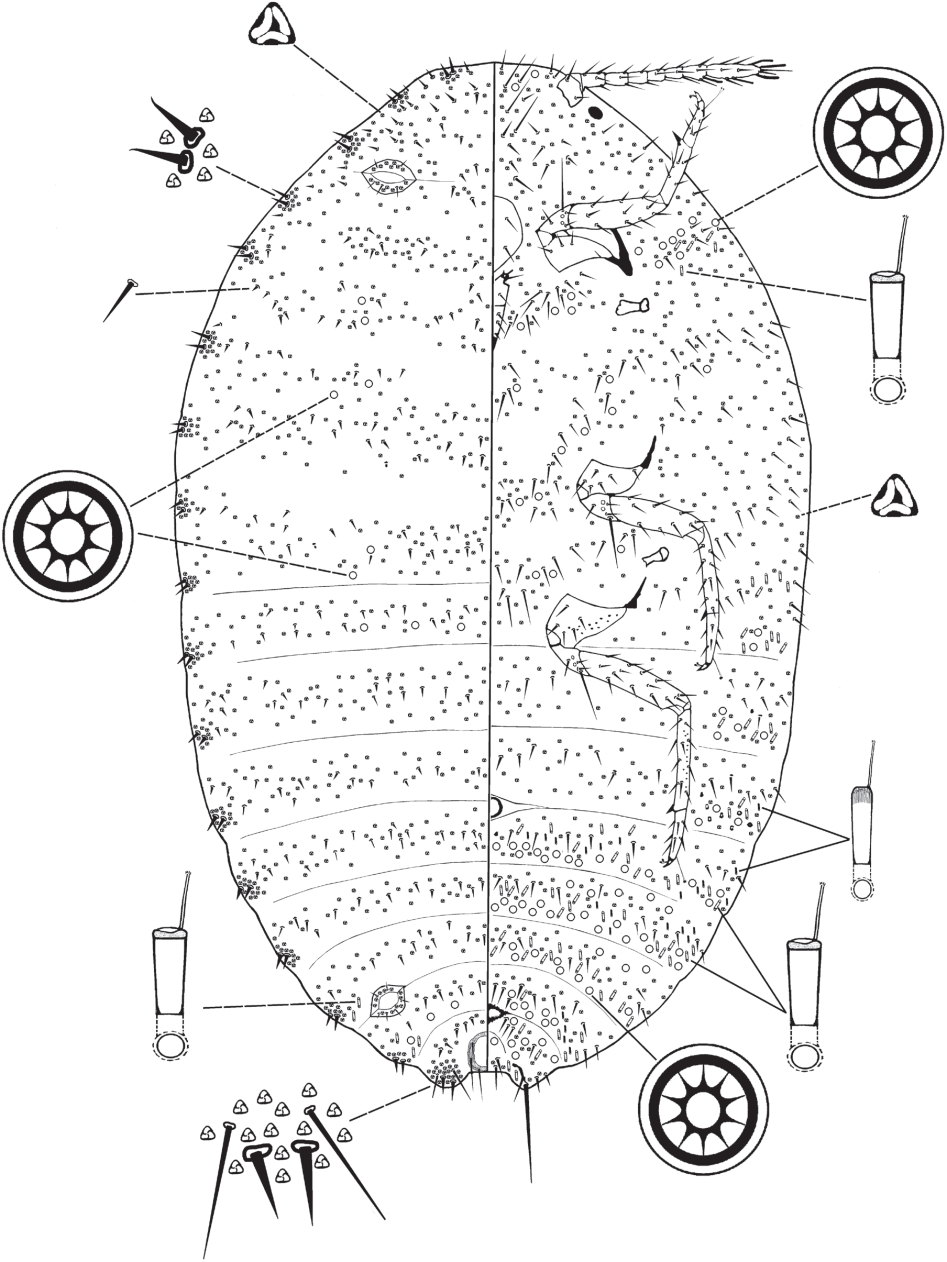
Adult female of *Planococcusbagmaticus* Williams. Specimen from Indonesia.

#### ﻿ Planococcus bendovi

Taxon classificationAnimaliaHemipteraPseudococcidae

Williams

745A6B66-EFC8-53D1-9939-750AEA094539

##### Material examined.

INDONESIA, Sumatra, Bengkulu Province, Bengkulu City, Muara Bangkahulu, on *Loranthus* sp. (Loranthaceae), living on cucumber tree (*Averrhoabilimbi* L.), 30 m a.s.l., 03°45'36″S, 102°16'01″E, 12.vi.2022, coll. A. Zarkani (ΑΖ1080-1081), 3 ♀♀.

##### Comments.

The holotype of *P.bendovi* was collected on peanut, *Arachishypogaea* L. (Fabaceae) in Tripura and Orissa, India (Williams 2004). However, in Indonesia, Zarkani et al. (2022) reported the species as being abundant on a semi-parasitic plant, *L.pentandrus*, living on avocado, cacao, citrus, and cucumber tree with an incidence rate up to 20–40%.

#### ﻿ Planococcus lilacinus

Taxon classificationAnimaliaHemipteraPseudococcidae

(Cockerell) *

A64DF3F6-956A-5320-8069-9980412F213A

##### Material examined.

Indonesia, Sumatra, Bengkulu Province, Bengkulu City, Slebar, on *Loranthus* sp. (Loranthaceae), living on avocado tree (*Perseaamericana* Mill.), 20 m a.s.l., 03°49'25″S, 102°19'08″E, 08.vii.2022, coll. A. Zarkani (AZ1118-1120), 6 ♀♀.

##### Comments.

The species is polyphagous on ornamental plants and fruit trees; it has been recorded from 36 plant families and 73 genera (García-Morales et al. 2016). It is cosmopolitan, having been reported from 34 countries (García-Morales et al. 2016). In Indonesia it is widely spread in Bali, Flores, Irian Jaya, Java, Kalimantan, Lombok and Sulawesi (Williams 2004; Zarkani et al. 2021).

### ﻿*Pseudococcus* Westwood

#### ﻿ Pseudococcus jackbeardsleyi

Taxon classificationAnimaliaHemipteraPseudococcidae

Gimpel & Miller*

C3B40A51-2C27-53B3-A7B4-B546C2D0AF96

##### Material examined.

Indonesia, Sumatra, Bengkulu Province, Seluma City, Air Periukan, on *Loranthus* sp. (Loranthaceae). living on cacao trees (*Theobromacacao* L.), 30 m a.s.l., 04°01'37″S, 102°24'50″E, 08.vii.2022, coll. A. Zarkani (AZ1112-1114), 6 ♀♀.

##### Comments.

The species is polyphagous on ornamental plants and fruit trees; it has been recorded from 54 plant families and 114 genera (García-Morales et al. 2016). It is cosmopolitan, having been reported from 54 countries (García-Morales et al. 2016). This is the first record for Sumatra, however, in Indonesia it has been recorded previously from Irian Jaya (Gavrilov-Zimin 2013), Flores (Gavrilov-Zimin 2017) and Java (Williams 2004).

## Supplementary Material

XML Treatment for Chorizococcus

XML Treatment for Chorizococcus ozeri

XML Treatment for Dysmicoccus lepelleyi

XML Treatment for Dysmicoccus zeynepae

XML Treatment for Ferrisia dasylirii

XML Treatment for Planococcus bagmaticus

XML Treatment for Planococcus bendovi

XML Treatment for Planococcus lilacinus

XML Treatment for Pseudococcus jackbeardsleyi

## ﻿Additional information

### Author ORCIDs

Agustin Zarkani https://orcid.org/0000-0001-9837-5019

Ariffatchur Fauzi https://orcid.org/0000-0001-9900-0614

Dwinardi Apriyanto https://orcid.org/0000-0002-5286-9010

Mehmet Bora Kaydan https://orcid.org/0000-0002-0677-255X

### Data availability

All of the data that support the findings of this study are available in the main text or Supplementary Information.
